# Protein Quality Control by Molecular Chaperones in Neurodegeneration

**DOI:** 10.3389/fnins.2017.00185

**Published:** 2017-04-06

**Authors:** Aaron Ciechanover, Yong Tae Kwon

**Affiliations:** ^1^Department of Biomedical Sciences, Protein Metabolism Medical Research Center, College of Medicine, Seoul National UniversitySeoul, South Korea; ^2^Technion Integrated Cancer Center, Rappaport Faculty of Medicine and Research Institute, Technion-Israel Institute of TechnologyHaifa, Israel; ^3^Ischemic/Hypoxic Disease Institute, College of Medicine, Seoul National UniversitySeoul, South Korea

**Keywords:** proteolysis, protein aggregation, ubiquitin-proteasome system, autophagy-lysosome system, chaperon-mediated autophagy, macroautophagy, protein quality control

## Abstract

Protein homeostasis (proteostasis) requires the timely degradation of misfolded proteins and their aggregates by protein quality control (PQC), of which molecular chaperones are an essential component. Compared with other cell types, PQC in neurons is particularly challenging because they have a unique cellular structure with long extensions. Making it worse, neurons are postmitotic, i.e., cannot dilute toxic substances by division, and, thus, are highly sensitive to misfolded proteins, especially as they age. Failure in PQC is often associated with neurodegenerative diseases, such as Huntington's disease (HD), Alzheimer's disease (AD), Parkinson's disease (PD), and prion disease. In fact, many neurodegenerative diseases are considered to be protein misfolding disorders. To prevent the accumulation of disease-causing aggregates, neurons utilize a repertoire of chaperones that recognize misfolded proteins through exposed hydrophobic surfaces and assist their refolding. If such an effort fails, chaperones can facilitate the degradation of terminally misfolded proteins through either the ubiquitin (Ub)-proteasome system (UPS) or the autophagy-lysosome system (hereafter autophagy). If soluble, the substrates associated with chaperones, such as Hsp70, are ubiquitinated by Ub ligases and degraded through the proteasome complex. Some misfolded proteins carrying the KFERQ motif are recognized by the chaperone Hsc70 and delivered to the lysosomal lumen through a process called, chaperone-mediated autophagy (CMA). Aggregation-prone misfolded proteins that remain unprocessed are directed to macroautophagy in which cargoes are collected by adaptors, such as p62/SQSTM-1/Sequestosome-1, and delivered to the autophagosome for lysosomal degradation. The aggregates that have survived all these refolding/degradative processes can still be directly dissolved, i.e., disaggregated by chaperones. Studies have shown that molecular chaperones alleviate the pathogenic symptoms by neurodegeneration-causing protein aggregates. Chaperone-inducing drugs and anti-aggregation drugs are actively exploited for beneficial effects on symptoms of disease. Here, we discuss how chaperones protect misfolded proteins from aggregation and mediate the degradation of terminally misfolded proteins in collaboration with cellular degradative machinery. The topics also include therapeutic approaches to improve the expression and turnover of molecular chaperones and to develop anti-aggregation drugs.

## Introduction

Proteins may lose their folding when cells are exposed to stresses, such as oxidative stress, heat, and toxic chemicals. Misfolded proteins and their aggregates grow into intracellular or extracellular amyloid plaques or neurofibrillary tangles (Taylor et al., 2002). Eukaryotic cells operate the PQC system to remove these cytotoxic agents in a timely fashion. The excessive formation of protein aggregates and their fibrillar structures are universally observed in at least 30 different human diseases (Taylor et al., 2002; Broersen et al., 2010). These protein misfolding disorders include various neurodegenerative diseases, such as Alzheimer' disease (AD), Parkinson' disease (PD), Huntington disease' (HD), transmissible spongiform encephalopathies (TSE), and amyotrophic lateral sclerosis (ALS) (Moreno-Gonzalez and Soto, 2011; Doyle et al., 2013; Hetz and Mollereau, 2014; Valastyan and Lindquist, 2014).

One essential component of PQC is molecular chaperones that enhance the refolding of misfolded proteins and, thus, counteract their aggregation (Hartl et al., 2011; Kim et al., 2013). Molecular chaperones constitute up to 10% of the proteome and play important functions in proteostasis under normal conditions as well as during cellular stress responses (Kastle and Grune, 2012). The majority of molecular chaperones are called heat-shock proteins (HSPs) because they are induced by various stresses such as heat shock, oxidative stress, toxic chemical, and inflammation (Garrido et al., 2001). HSPs are divided into several subgroups based on their sizes, such as Hsp70, Hsp90, Hsp60, Hsp40 (DnaJ), and small HSPs. These molecular chaperones can assist the refolding of misfolded proteins through three distinct action modes. *First*, most chaperones such as Hsp70 can hold the clients in an unfolded state until spontaneous fold is achieved (Rudiger et al., 1997; Hartl et al., 2011; Kastle and Grune, 2012). *Second*, some molecular chaperons such as Hsp70 and Hsp60s can use ATP to unfold stable misfolded proteins and convert them into natively refoldable species (Ranford et al., 2000; Itoh et al., 2002; Tutar and Tutar, 2010). *Third*, some chaperones, such as yeast Hsp104 and human Hsp70 in complex with Hsp40 and Hsp110, can act as “disaggregases” because they use the energy of ATP hydrolysis to forcefully unfold and solubilize preformed aggregates into natively refolded proteins (Mosser et al., 2004; Shorter and Lindquist, 2004; Arimon et al., 2008; Lo Bianco et al., 2008; DeSantis et al., 2012). Despite distinct action modes, they share general properties to recognize and bind the hydrophobic sequences which are not normally exposed in the native folding (Buchner, 1996). Their binding to and dissociation from clients can be driven by adenosine-5′-triphosphate (ATP) hydrolysis. The ATPase and chaperone activity are typically regulated through their cooperation with cochaperones. In addition to ATP-dependent chaperones, neurons express ATP-independent chaperons that bind misfolded proteins and promote refolding (D'Andrea and Regan, 2003). These chaperones typically form a coordinated network with cochaperones and the machinery in proteolytic pathways.

While the primary function of molecular chaperones is to assist misfolded or unfolded proteins to regain or acquire the normal folding, they can facilitate the degradation of terminally misfolded proteins in collaboration with proteolytic machinery (Hoffmann et al., 2004; Ellis, 2006, 2007; Ellis and Minton, 2006; Pauwels et al., 2007). Eukaryotic cells operate two major proteolytic systems, the UPS and autophagy. In principle, terminally misfolded proteins are ubiquitinated by E3 Ub ligases and processively degraded by the proteasome. If the substrates are prone to aggregation or escape the surveillance of the UPS, however, they are redirected to macroautophagy in which cargoes are separated in the double membrane structure, called the autophagosome, and degraded by lysosomal hydrolases (Cha-Molstad et al., 2015). Some misfolded proteins carrying the KFERQ pentapeptide sequence can be sorted out by molecular chaperones and directly delivered to the lysosome through chaperone-mediated autophagy (CMA) (Chiang et al., 1989; Dice, 1990; Cuervo et al., 1997).

The UPS is an intracellular proteolytic system that mediates the degradation of more than 80% of normal and abnormal intracellular proteins (Wang and Maldonado, 2006). The importance of molecular chaperones in the UPS was initially proposed and demonstrated by Ciechanover and colleagues who showed that the molecular chaperone Hsc70 is required for Ub-dependent degradation of several substrates (Ciechanover et al., 1995; Bercovich et al., 1997). The UPS involves a cascade of E1, E2, and E3 enzymes whose cooperative activities mediate the conjugation of Ub to target proteins (Pickart, 2001). In this cascade, Ub with a size of 76 residues is activated by the Ub activating enzyme E1 and transferred to the Ub conjugating enzyme E2. The Ub moiety carried by E2 is conjugated to substrates, which requires the ubiquitination activity of the Ub ligase E3. In PQC, most E3s cannot recognize misfolded proteins and rather depend on molecular chaperones for substrate recognition. Ubiquitinated substrates are degraded by the proteasome into short peptides, typically with sizes of 8–12 amino acids. These peptides are displayed on the cell surface for immunosurveillance (Kloetzel and Ossendorp, 2004) or degraded into free amino acids by aminopeptidases. The UPS plays a pivotal role in proteostasis during neurodegeneration and prevents protein misfolding and aggregation (Morawe et al., 2012). In addition to PQC, the UPS regulates a variety of biological processes, including cell cycle, transcription, DNA repair, and apoptosis (Eldridge and O'Brien, 2010; Xie, 2010).

Autophagy is a process by which cytosolic materials are degraded by the lysosome. Depending on the mechanism of cargo delivery to the lysosome, autophagy can be divided into three pathways: microautophagy, CMA, and macroautophagy. In macroautophagy, terminally misfolded proteins in complex with molecular chaperones are collected by autophagy adaptors, such as p62 and NBR1. Cargo-loaded p62 undergoes self-polymerization and are deposited to the autophagosome through the interaction of p62 with LC3 (Lamark et al., 2009; Stolz et al., 2014). The autophagosome is fused with the lysosome to form the autolysosome wherein cargoes along with p62 are degraded by lysosomal hydrolases. Virtually all the misfolded proteins including those prone to aggregation in neurodegenerative diseases can be degraded by macroautophagy. In contrast to macroautophagy, CMA targets a subset of misfolded cytosolic proteins, especially those containing the KFERQ pentapeptide sequence (Fuertes et al., 2003; Massey et al., 2006; Kaushik and Cuervo, 2012). The substrates of the CMA are recognized by the molecular chaperone Hsc70 belonging to the Hsp70 family (Chiang et al., 1989). The cargo-Hsc70 complex is translocated into the lysosomal lumen and degraded by lysosomal hydrolases (Cuervo and Dice, 1996). Overall, lysosomal proteolysis through macroautophagy and CMA plays an important role in the removal of misfolded proteins that cannot be readily degraded by the UPS.

Misfolded proteins that survive the attempts of molecular chaperones to refold or degrade eventually form aggregates. As the last defense mechanism of PQC, molecular chaperons can directly resolve, i.e., disaggregate the already formed aggregates (Parsell et al., 1994; Mogk et al., 1999; Doyle et al., 2013). The disaggregation activity has been characterized in yeasts and mammals (Weibezahn et al., 2005; Hodson et al., 2012; Winkler et al., 2012). In yeasts, Hsp104 in collaboration with Hsp70, Hsp40, Hsp110, and sHSPs can directly disaggregate and reactivate proteins deposited in high order aggregates (Shorter, 2011; Torrente and Shorter, 2013). In mammals, Hsp110, Hsp105, Hsp100, and Hsp70/Hsp40 have been implicated in disaggregation (Lindquist and Kim, 1996; Glover and Lindquist, 1998; Doyle and Wickner, 2009). Through these multi-step defense processes, molecular chaperones play a key role in proteostasis.

Recent studies using mouse models suggest that molecular chaperones play a protective role in the pathogenesis of neurodegenerative disorders (Wyatt et al., 2012; Carman et al., 2013; Witt, 2013). By using disease models, HSPs have been shown to inhibit the aggregation of aggregation-prone proteins, such as Aβ, tau, HTT, and α-synuclein, and facilitate their degradation by the UPS or autophagy (Wyttenbach, 2004). As such, small molecule compounds that can modulate HSPs and proteolytic machinery are emerging as a means to treat neurodegenerative diseases. Below, we discuss the current understanding on the functions of HSPs in neurodegenerative diseases, including the recent results obtained from animal models of neurodegeneration.

## Refolding of misfolded proteins by molecular chaperones

Neurons express various molecular chaperones which forms a complicated network of PQC to prevent aggregation. Their primary function is to assist the folding and assembly of newly synthesized polypeptides and the refolding of misfolded or damaged proteins. Depending on their sizes and action modes, molecular chaperones can be divided into several classes based on their sizes, such as Hsp70, Hsp90, Hsp60, Hsp40 (DnaJ), and small HSPs. Although the majority of molecular chaperones share similarity in action modes, such as substrate recognition and ATP hydrolysis-driven substrate binding, they are also different in substrate specificity, localization, and mechanistic details.

### The Hsp70 family

The cytosolic chaperone Hsp70 is evolutionarily conserved and one of the most abundant chaperones. The homologs of Hsp70 are found in various subcellular compartments, including heat shock cognate 70 (Hsc70) in the cytosol and BiP/GRP78 in the ER. Hsp70 shows a broad range of activities in folding newly synthesized polypeptides, refolding misfolded proteins, the degradation of terminally misfolded proteins, and directly resolving already formed aggregates (Kastle and Grune, 2012). They commonly recognize diverse misfolded proteins through the interaction with a four to five residue stretch of hydrophobic amino acids exposed on the surface (Rudiger et al., 1997). The hydrophobic signatures occur on average every 30–40 residues in most misfolded proteins. Central to the chaperone activity of Hsp70 proteins is the transition between open and closed conformations of their substrate binding domain (SBD). In the ATP-bound open conformation, the SBD has low affinity to the client (Hartl et al., 2011). Once ATP hydrolysis is induced by cochaperones, Hsp70 acquires high affinity to the clients. The resulting ADP-bound form of Hsp70 facilitates the client' refolding by holding them in an unfolded state until spontaneous fold is achieved. The client that achieved the correct folding no longer has the exposed hydrophobic patches and, thus, is released from Hsp70. Extensive studies have shown that Hsp70 directly binds various pathogenic misfolded proteins in neurodegenerative diseases and facilitate their refolding. Such substrates of Hsp70 proteins include mutant huntingtin (mHTT) in HD and other polyQ diseases, α-synuclein in PD, amyloid-β (Aβ) and hyperphosphorylated tau in AD, and mutant SOD1 in ALS (Choo et al., 2004; Dedmon et al., 2005; Liu et al., 2005; Evans et al., 2006; Dompierre et al., 2007; Luk et al., 2008).

### The Hsp40 (DnaJ) family

The Hsp40 proteins, also called J-proteins, form a large cochaperone family composed of 49 members (Odunuga et al., 2003). Amongst these, DnaJB6 and DnaJB8 are mainly expressed in neurons and can suppress polyglutamine aggregation and toxicity (Cheetham et al., 1992; Hageman et al., 2010). Although these cochaperones have the activity to bind and counteract protein aggregates or refold them, they also can modulate the ATP hydrolysis of Hsp70. The 70-residue J domain of Hsp40 binds misfolded proteins and interacts with the ATPase domain of Hsp70, which induces the ATP hydrolysis of Hsp70. ATP hydrolysis, in turn, brings the Hsp40-bound substrate close to the SBD of Hsp70 and increases Hsp70 affinity to the substrate, leading to Hsp40 release from the substrate and Hsp70 (Summers et al., 2009). As a consequence of this allosteric conformational change, the substrate is transferred from Hsp40 to Hsp70. Besides the conserved J domain, Hsp40 proteins carry diverse domains that mediate specific biological processes, such as intracellular localizations and client binding for proteolysis (Cheetham and Caplan, 1998; Kampinga and Craig, 2010). In neurodegenerative disease, Hsp40 proteins can act as cochaperones for Hsp70 proteins to assist the refolding of soluble misfolded proteins (Choo et al., 2004; Dedmon et al., 2005; Liu et al., 2005; Evans et al., 2006; Dompierre et al., 2007; Luk et al., 2008).

### The Hsp90 family

The ATP-dependent chaperone Hsp90, which forms a dimer, is universally present in various cellular compartments, such as the cytosol, nucleus, ER, and mitochondria (Lindquist, 2009). Hsp90 is constitutively expressed in normal conditions, accounting for 1–2% of cellular proteins, and its level can increase to 4–6% if cells are exposed to stresses (Picard, 2002; Whitesell and Lindquist, 2005; Taipale et al., 2010; Finka and Goloubinoff, 2013). The activity of Hsp90 can be regulated by the HSR (heat-shock response) regulator HSF1 (heat-shock factor 1) (McLean et al., 2004; Putcha et al., 2010). Human neurons have a stress-inducible Hsp90α (Hsp90AA1) and a constitutively expressed Hsp90β (Hsp90AB1) that share 86% identity in protein sequence (Ammirante et al., 2008). These Hsp90 proteins bind a variety of clients and hold their folding, including kinases, nuclear receptors, transcription factors and cell surface receptors (Kastle and Grune, 2012). Remarkably, Hsp90 is thought to interact with approximately 2,000 proteins (Garnier et al., 2002), which accounts for up to 10% of total cellular proteins (Ratzke et al., 2010). Structural studies have shown that Hsp90 is composed of an N-terminal ATP-binding domain (N-domain), a mid-domain that binds the substrate (M-domain), and a C-terminal dimerization domain (C-domain) (Picard, 2002; Whitesell and Lindquist, 2005; Taipale et al., 2010; Finka and Goloubinoff, 2013). The substrate binding-release cycle of Hsp90 is regulated by ATP hydrolysis, which induces a large conformational transition between an open vs. closed form. In a free form, Hsp90 is in an open V-shaped conformation and, thus, binds clients. The ATP binding to the N-domain of client-loaded Hsp90 induces a conformational transition (Pearl and Prodromou, 2006). This results in a closed conformation where the N-domains of two Hsp90 molecules dimerize with each other. Following ATP hydrolysis, the substrate is released, and Hsp90 returns to an open conformation. The conformational transition of Hsp90 is regulated by various cochaperones, such as Hop, p23/Sba1, and Cdc37 (Picard, 2002; Whitesell and Lindquist, 2005; Taipale et al., 2010; Finka and Goloubinoff, 2013). Overall, the ability of Hsp90 to support the folding/refolding and stability of proteins is a double edge blade in neurodegeneration because it can also favor the accumulation of toxic protein aggregates (Schulte and Neckers, 1998; Boland et al., 2008; Eskelinen and Saftig, 2009; Chouraki and Seshadri, 2014).

### The Hsp60 family

Hsp60, also called chaperonins, is a 60 kDa mitochondrial chaperone (Ranford et al., 2000; Itoh et al., 2002; Tutar and Tutar, 2010). GroEL, a well characterized bacterial chaperone, also belongs to this class. Although the primary location of Hsp60 is mitochondria, it can migrate to the cytosol under certain cellular stresses (Ranford et al., 2000; Itoh et al., 2002; Tutar and Tutar, 2010). Hsp60 forms a double ring complex, in which each rich is composed of seven subunits. Clients are fed into the central cavity of the double ring complex, in which their exposed hydrophobic residues are sequestered during refolding process (Ranford et al., 2000; Tutar and Tutar, 2010). The folding process by Hsp60 is modulated by a lid, which are formed by cochaperones such as Hsp10 in mitochondria. Following ATP hydrolysis, the unfolded client is released through the opening of the Hsp10 lid, now with a native folding (Ranford et al., 2000; Tutar and Tutar, 2010). Hsp60 works together with Hsp70 for protein folding of unfolded proteins. Neurons contain another type of chaperonins in the cytosol, which do not depend on cochaperones. They form a homotypic or heterotypic double ring complex, each of which is composed of eight subunits. The members of this group include the TCP-1 Ring Complex (TRiC), alternatively called TCP1 complex (CCT) (Lopez et al., 2015). Although, studies have shown that Hsp60 interacts with mutant α-synuclein in PD (Irizarry et al., 1998; Spillantini et al., 1998), the physiological importance of Hsp60 proteins in the refolding of pathogenic misfolded proteins in neurodegeneration remains poorly characterized.

### The small HSP family

Different from other types of HSPs, small HSPs are ATP independent. To date, 10 small HSPs with sizes ranging from 12 to 42 kDa are known in humans. In mouse brain, five small HSPs are prominently expressed (Quraishe et al., 2008). Amongst these, the neuronal expression of Hsp27 and αB crystallin is selectively induced under stresses (Quraishe et al., 2008). Members of this family are characterized by a 100-residue α-crystallin domain flanked by variable N-terminal and C-terminal extensions. These extensions are responsible for substrate recognition and mediates the formation of oligomers. As holding factors, small HSPs bind to unfolded or misfolded proteins and prevent their aggregation until the clients are delivered to other chaperones, such as Hsp70 and Hsp40 system (Carra et al., 2012). Amongst these, Hsp27 is the most abundant and well characterized. Their expression is selectively induces by various stresses that perturb proteostasis (Sarto et al., 2000; Sun and MacRae, 2005).

## Degradation of misfolded proteins by molecular chaperones through the UPS

While the primary functions of molecular chaperones relate refolding and unfolding of nascent and misfolded proteins, they can facilitate the degradation of terminally misfolded clients, either through the UPS or autophagy (Lanneau et al., 2010). The majority of these clients are tagged with Ub chains for degradation by the proteasome complex. However, the substrates prone to aggregation are redirected to autophagy. In either case, molecular chaperones are involved in the recognition and/or delivery of terminally misfolded substrates.

### Ub-dependent selective proteolysis through the proteasome

The UPS mediates selective proteolysis of short-lived proteins by the proteasome and accounts for more than 80% of intracellular proteolysis (Hershko and Ciechanover, 1998). In the UPS, Ub is first activated by its conjugation to the ubiquitin activating enzyme E1. This conjugation involves the ATP-dependent formation of a thioester bond between the C-terminal glycine residue of Ub and an active site cysteine of E1 (Hershko and Ciechanover, 1998; Ciechanover, 2013). The activated Ub is transferred to the Ub conjugating enzyme E2 through a thioester bond. The Ub ligase E3 promotes the transfer of Ub from E2 to the lysine (Lys) residue of substrates. This generates an isopeptide bond between the C-terminal glycine residue of Ub and lysine residues on the substrate (Hershko and Ciechanover, 1998; Ciechanover, 2013). The human genome encodes more than 500 E3s. These E3s can be divided into three groups depending on their ubiquitination domains, including the really interesting new gene (RING) finger, the homologous to E6-AP (HECT) domain, and the U-box domain (Qian et al., 2006). Occasionally, E4 enzymes enhance the conjugation of additional Ub molecules to form a Ub chain, typically through the K48 linkage (Upadhya and Hegde, 2007). Once the first Ub is attached to substrate, subsequent Ub conjugations may use any of its seven Lys residues (Peng et al., 2003). This can generates a Ub chain with many different topologies, each of which has distinct functions. Amongst these topologies, the most widely used Lys48 linkage typically leads to proteasomal degradation. The Lys63 linkage mediates nonproteolytic processes, such as Ub-dependent protein-protein interactions (Hadian et al., 2011). Human cells also use the Lys11 linkage for cell cycle regulation and cell division as well as ERAD (Matsumoto et al., 2010) and K27 for ubiquitin fusion and degradation (Morawe et al., 2012). Ub moieties conjugated to substrates are reversible and can be detached and adjusted by deubiquitination enzymes (DUBs). The substrates conjugated with polyubiquitin are degraded by the 26S proteasome (Hershko and Ciechanover, 1998). This 2.5-MDa protease complex is composed of the 20S core particle with a size of 700 kDa associated with two 19S regulatory particles (Ravikumar et al., 2008; Douglas et al., 2009; Ciechanover and Kwon, 2015). The Ub chains conjugated to substrates are recognized by RPN10 and RPN13 of the 19S particle and stripped off by DUBs such as RPN11, USP14, and UCHL5 (ubiquitin C-terminal hydrolase L5; Ravikumar et al., 2008; Douglas et al., 2009; Ciechanover and Kwon, 2015). Deubiquitinated substrates are unfolded into a nascent polypeptide through ATP hydrolysis in the 19S particle and fed into the 20S particle for degradation, generating short peptides with average sizes of 8–12 amino acids (Hershko and Ciechanover, 1998). These peptides are degraded into amino acids by aminopeptidases, which are recycled for protein synthesis, or presented on the cell surface for immunosurveillance (Kloetzel and Ossendorp, 2004).

### Molecular chaperones and Ub ligases work together in the UPS

**Figure 1:**
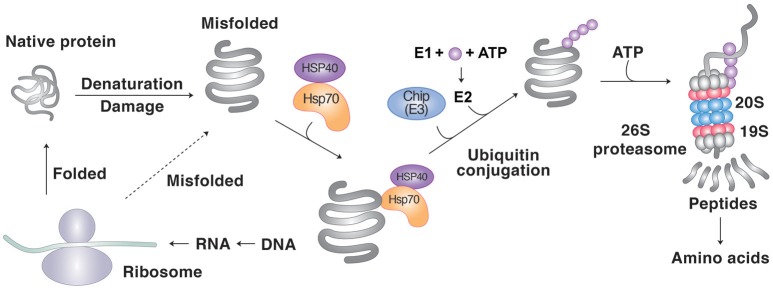
**Protein degradation by molecular chaperones through the UPS**. Molecular chaperones such as Hsp70 recognizes the hydrophobic sequences of misfolded proteins as degrons. The Ub ligase CHIP guides the chaperone-client complexes to the UPS and mediates the clients' ubiquitination. The UPS involves a cascade of E1, E2, and E3 enzymes whose cooperative activities mediate the conjugation of Ub to target proteins. In PQC, most E3s cannot recognize misfolded proteins and rather depend on molecular chaperones for substrate recognition. Ubiquitinated substrates are degraded by the proteasome into short peptides, typically with sizes of 8–12 amino acids.

Several cytosolic or nuclear Ub ligases are known to be involved in degradation of misfolded proteins in collaboration with molecule chaperones, including UBR1, UBR2, San1, Hul5, E6-AP, C-terminus of Hsc70-interacting protein (CHIP) and Parkin (Gardner et al., 2005; Heck et al., 2010; Kettern et al., 2010; Figure 1). CHIP is a 35 kDa protein that has dual functions, one as a cochaperone of Hsp70 and Hsp90, and the other as a Ub ligase that mediates ubiquitination of misfolded proteins using its the RING-like U-box domain (Ballinger et al., 1999; McDonough and Patterson, 2003). The E3 activity of CHIP requires the interaction with the E2 Ub conjugating enzyme UBCH5 (Cyr et al., 2002). When interacting with the Hsp70- or Hsp90-client complex, CHIP in collaboration with UBCH5 captures and ubiquitinates the misfolded clients for proteasomal degradation (Demand et al., 2001). To facilitate the delivery of the ubiquitinated substrates to the proteasome, CHIP also interacts with the S5a component (also known as Rpn10) of the 19S proteasome particle (Connell et al., 2001). In this process, CHIP indirectly recognize misfolded proteins through the interaction between its TPR (tetratricopeptide repeat) domain with Hsp70 or Hsp90 (Lanneau et al., 2010). The substrates of CHIP include hyperphosphorylated tau and mutant SOD1 (Lanneau et al., 2010). CHIP-mediated degradation of Hsp70 clients is further facilitated by the cochaperone BAG-1 (Takayama et al., 1997). BAG-1 uses its C-terminal region to bind the ATPase domain of Hsp70 and acts as a nucleotide exchange factor (NEF), inducing the release of substrates from Hsp70 (Takayama et al., 1997). On the other hand, BAG-1 has also a Ub-like (UBL) domain at its N-terminal region that supports the interaction with the proteasome (Alberti et al., 2003). BAG-1 directly interacts and cooperates with CHIP to guide the terminally misfolded clients to the UPS. In addition to BAG-1, Hsp27 belonging to the small HSP family can directly interact with the proteasome to modulate the ubiquitination of clients (Garrido et al., 2006). Hsp27 also binds the Ub chain of clients and, thus, increase the degradation of ubiquitinated proteins (Garrido et al., 2006).

The clients of Hsp90 can also be degraded through the UPS if they are no longer chaperoned by Hsp90, for example, owing to misfolding. These misfolded clients dissociated form Hsp90 are ubiquitinated by E3 ligases, such as CHIP, and degraded by the proteasome (Didelot et al., 2007). However, CHIP is mainly associated with Hsp70, and there should be additional E3 ligases that target the misfolded clients of Hsp90. One such candidate is the E3 ligase Triad3A which forms a complex with Hsp90 and receptor interacting protein 1 (RIP-1) and mediates the ubiquitination of RIP-1 and proteasomal degradation following Hsp90 inhibition by geldanamycin (Fearns et al., 2006).

The N-end rule pathway is a proteolytic system in which a single N-terminal residue acts as an essential component of a class of degrons, called N-degrons (Bachmair et al., 1986; Tasaki and Kwon, 2007; Sriram and Kwon, 2010; Sriram et al., 2011; Varshavsky, 2011). In mammals, these N-terminal degrons are recognized by the N-recognin family, including UBR1, UBR2, UBR4, UBR5, and p62 (Kwon et al., 1999a, 2002; Tasaki et al., 2005, 2009; An et al., 2006). Amongst these, the Ub ligases UBR1 and UBR2 have been shown to mediate the ubiquitination of misfolded cytosolic proteins, leading to proteasomal degradation (Eisele and Wolf, 2008; Heck et al., 2010; Prasad et al., 2010). These RING finger E3 ligases indirectly recognize misfolded proteins through molecular chaperones such as Hsp110 and Hsp70 (Heck et al., 2010; Nillegoda et al., 2010). Misfolded proteins targeted by N-recognins include TDP43 in ALS and tau and amyloid β in AD (Brower et al., 2013). Interestingly, in addition to the exposed hydrophobic residues, some of their misfolded clients are post-translationally conjugated with the amino acid L-Arg of Arg-tRNA^Arg^ by *ATE1*-encoded R-transferases (Grigoryev et al., 1996; Balogh et al., 2000, 2001; Kwon et al., 2000; Lee et al., 2005). The resulting N-terminal Arg residue acts as N-degron which is recognized by N-recognins such as UBR1 and UBR2 (Kwon et al., 1999b; Lee et al., 2008; Sriram et al., 2009; Meisenberg et al., 2012). In yeasts, the cytosolic E3 ligase Ubr1 has been shown to work with the nuclear E3 ligase San1 if cytosolic misfolded proteins overwhelm the capacity of E3 ligases (Heck et al., 2010; Prasad et al., 2010). In this collaboration between cytosolic and nuclear PQC systems, San1 associated with Hsp70 brings excessive cytosolic misfolded proteins to the nucleus for proteasomal degradation (Heck et al., 2010; Prasad et al., 2010). Different from other E3 ligases, San1 has many disordered structures and stretches of hydrophobic residues and, thus, can directly bind misfolded proteins (Rosenbaum et al., 2011). In mammals, the nuclear Ub ligase UHRF2 has been proposed to be a functional homolog of the yeast San1 (Nielsen et al., 2014).

Eukaryotic cells operate various degradative machinery designated to specific types of misfolded proteins. In yeasts, misfolded proteins generated by heat shock are specifically ubiquitinated by the E3 ligase Hul5 that has a HECT ubiquitination domain (Fang et al., 2011). In mammals, mislocalized membrane proteins are ubiquitinated by the Ub ligase RNF126 (RING finger 126) in collaboration with the BAG6 chaperone system (Rodrigo-Brenni et al., 2014). Proteins synthesized from aberrant mRNAs without stop codons are ubiquitinated by Listerin/Ltn1 (Bengtson and Joazeiro, 2010). Moreover, in the ER, membrane-associated misfolded proteins are ubiquitinated by the Ub ligase DOA10 (Nielsen et al., 2014). By contrast, misfolded proteins in the ER lumen are ubiquitinated by the Ub ligases Hrd1 and Gp78 mediates through a process called ERAD (ER-associated degradation) (Vembar and Brodsky, 2008). Except for San1 and Hul5, most of these E3s indirectly recognize misfolded proteins through cooperating molecular chaperones. Overall, the mechanistic details and clinical importance of these various PQC machinery in neurodegenerative diseases remains largely unexplored.

### Deubiquitination enzymes (DUBs) in the degradation of misfolded proteins

DUBs detach Ub molecules from substrates and, thus, can modulate the proteasomal degradation of Ub-conjugated substrates. The proteasome is associated with DUBs such as RPN11, UCHL5, and USP14. RPN11 is a stoichiometric subunit of the proteasome and detaches Ub molecules *en bloc* from substrates (Hao et al., 2013). The free, unanchored Ub chains are deposited to aggresomes and recognized by HDAC which brings misfolded protein aggregates to aggresomes (Hao et al., 2013). The interaction between HDAC and unanchored Ub chains is essential for cargo-loaded HDAC to see where aggresomes are (Hao et al., 2013). In contrast to RPN11, USP14 is a conditionally recruited to the proteasome through its UBL domain. This enhances its activity up to 800-folds and, thus, modulates the degradation rate of substrates (Crosas et al., 2006). The treatment of the USP14 inhibitor IU1 has been shown to facilitate the degradation of aggregation-prone misfolded proteins such as tau and polyQ-expanded mutant ataxin-3 (Lee et al., 2010). The functions of DUBs in neurodegenerative diseases remain largely unexplored.

### The UPS is impaired during neurodegeneration

The pathogenesis of most neurodegenerative diseases, such as AD, PD, ALS, HD, and prion diseases commonly involves the downregulation of the components of the UPS. One prominent risk factor is aging. The activities of UPS components, such as the proteasome, are often progressively declined as neurons age (Keller et al., 2000; Hwang et al., 2007; Tydlacka et al., 2008; Low, 2011). This may reduce the ability to degrade misfolded proteins, contributing to the accumulation of pathological protein aggregates. Making it worse, the accumulated aggregates as a consequence of reduced UPS activities now further inhibit the activities of UPS components, including the proteasome. The proteasome is particularly vulnerable to protein aggregates because its narrow chamber has a diameter of as small as 13 angstroms. Therefore, proteasome cannot digest protein aggregates that cannot be easily unfolded. For example, β-sheet-rich PrP aggregates were shown to block the opening of the 20S proteasome particle, further reducing proteasomal activity (Andre and Tabrizi, 2012). Following ubiquitination and aggregation, tau in AD binds the recognition site of the 19S catalytic particle and block its gate (Dantuma and Lindsten, 2010; Tai et al., 2012). Aggregates of many other pathogenic proteins in neurodegenerative disorders can directly inhibit proteasome activity (Gregori et al., 1995; Snyder et al., 2003; Lindersson et al., 2004; Kristiansen et al., 2007). The resulting proteotoxicity has adverse effects on neurons (Hegde and Upadhya, 2011). Indeed, the reduced UPS activity has been associated with neuronal damage in AD, HD, PD, ALS, ataxia, Angelman syndrome, Wallerian degeneration, and gracile axonal dystrophy (Hegde, 2010; Hegde and Upadhya, 2011).

## Degradation of misfolded proteins by autophagy

**Figure 2:**
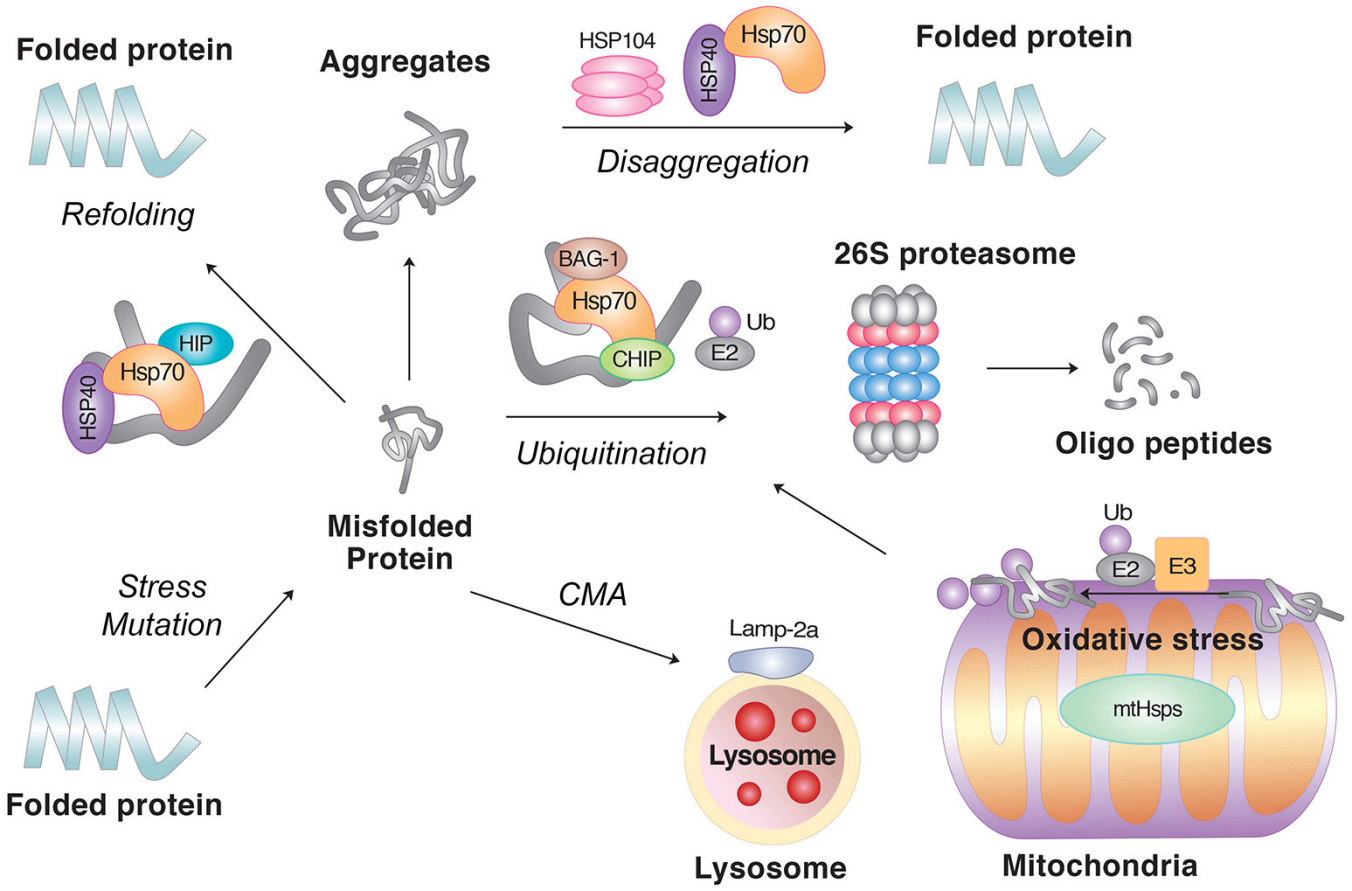
**The role of molecular chaperones in PQC**. Molecular chaperones, such as Hsp70 in combination with the cochaperone Hsp40, facilitate the refolding of misfolded proteins. If the clients fail to refold, molecular chaperones can also mediate their degradation in collaboration with cellular proteolytic pathways. In principle, soluble misfolded proteins are targeted by the UPS, in which the clients are ubiquitinated by E3 Ub ligases followed by degradation through the 26S proteasome. However, if the clients are prone to aggregation or escape the surveillance of the UPS, they can be degraded by lysosomal hydrolases, either through macroautophagy or CMA. As the last step of PQC, molecular chaperones can disaggregate already formed aggregates. Also shown are misfolded proteins induced by oxidative stress in mitochondria.

Autophagy is a process by which cytosolic materials are degraded by the lysosome. Depending on the mechanism of cargo delivery to the lysosome, autophagy can be divided into three pathways: microautophagy, CMA, and macroautophagy. Terminally misfolded proteins in neurodegenerative diseases can be degraded through macroautophagy or CMA (Figure 2). The role of autophagy in proteostasis is vitally important for postmitotic neurons with long extensions, in which cytotoxic proteins cannot be diluted by cell division.

### Macroautophagy

Misfolded proteins prone to aggregation can be directed to macroautophagy for lysosomal degradation. These substrates, typically as a Ub-conjugated form, are collected by autophagy adaptors, such as p62 and NBR1 (Cha-Molstad et al., 2015). P62 is normally inactive and can be activated by binding to the N-terminally arginylated form of the molecular chaperone BiP/GRP78, the ER counterpart of cytosolic Hsp70 (Cha-Molstad et al., 2015). Upon the accumulation of non-degradable autophagic cargoes, BiP and other ER-residing chaperones, such as calreticulin and protein disulfide isomerase (PDI), are N-terminally arginylated by *ATE1*-encoded R-transferase. The resulting N-terminally arginylated BiP, R-BiP, locates in the cytosol where R-BiP binds the ZZ domain of p62 through its N-terminal arginine residue. This binding induces a conformational change in p62, facilitating the polymerization of p62 as well as the interaction of p62 with LC3-II which is anchored on the membrane of autophagosomes (Cha-Molstad et al., 2015). It is generally assumed that p62 and other autophagic adaptors recognize the Ub moieties conjugated to misfolded proteins and delivers them to the autophagosome through specific interaction with LC3 (Lamark et al., 2009; Stolz et al., 2014). The cargo-loaded autophagosome is fused with the lysosome to form the autolysosome wherein cargoes along with p62 are degraded by lysosomal hydrolases.

### CMA

**Figure 3:**
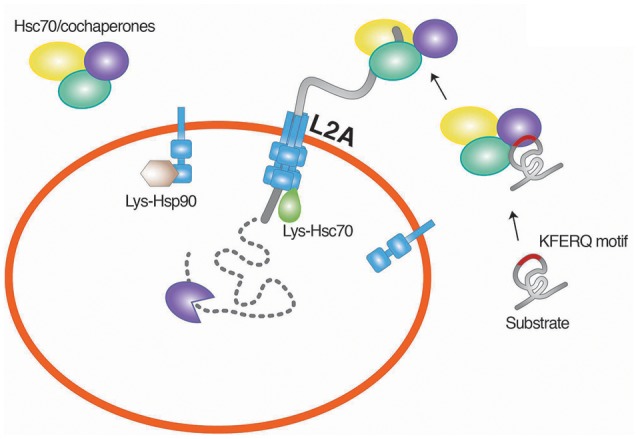
**Chaperone-mediated degradation of misfolded proteins**. CMA is a selective proteolytic system in which cytosolic proteins carrying the KFERQ pentapeptide are targeted by Hsc70. The function of Hsc70 requires cochaperones, such as Hsp40, Hsp90, HIP, HOP, and BAG-1. The substrates associated with the Hsc70 chaperone system are translocated to the lysosomal membrane through the interaction of Hsc70 with LAMP2A, a single-span membrane protein. L2A, LAMP2A.

CMA is a selective proteolytic system and does not involve vesicle formation (Chiang et al., 1989; Dice, 1990; Cuervo et al., 1997). The selectivity enables the degradation of misfolded or damaged cytosolic proteins without interfering with the same kinds of proteins with normal functions (Fuertes et al., 2003; Massey et al., 2006; Kaushik and Cuervo, 2012). The target substrates of the CMA include cytosolic proteins that carry the KFERQ pentapeptide which functions as a degron. The CMA degron, found in approximately 30% of cytosolic proteins (Chiang and Dice, 1988; Dice, 1990), is recognized by chaperones associated with cochaperones such Hsc70 belonging to the Hsp70 family (Chiang et al., 1989) (Figure 3). The function of Hsc70 requires cochaperones, such as Hsp40, Hsp90, HIP, HOP, and BAG-1 (Agarraberes and Dice, 2001). The substrates associated with the Hsc70 chaperone system are translocated to the lysosomal membrane through the interaction of Hsc70 with LAMP2A, a single-span membrane protein (Cuervo and Dice, 1996). The stability of LAMP2A requires its association with Lys-Hsc70, a lysosomal homolog of Hsc70. Once the substrate is targeted to the lysosome, Lys-Hsc70 assists the active LAMP2A complex to be disassembled into the inactive monomeric form, which is now available for the next round of the CMA process (Bandyopadhyay et al., 2008). The levels of LAMP2A and Lys-Hsc70 are important underlying the rate of CMA degradation (Cuervo et al., 1995; Agarraberes et al., 1997; Cuervo and Dice, 2000). Although a large number of cytosolic proteins contain the CMA degron sequence, only a limited number of these proteins were demonstrated to be degraded by the CMA (Wing et al., 1991). Post-translational modifications can generate the substrates of the CMA (Chiang and Dice, 1988; Dice, 1990). Studies have shown that CMA is essential for the survival of neurons by degrading misfolded or damaged cytosolic proteins (Cuervo et al., 2004). The misregulation of the CMA has been shown to correlate to the pathogenesis of neurodegeneration (Cuervo et al., 2004).

## Disaggregation of aggregates by molecular chaperones

Misfolded proteins may form aggregates if the PQC system is overwhelmed, for example, under severe stress conditions or by genetic mutations that allows the accumulation of non-degradable polypeptides. Yeast and mammalian cells have molecular chaperones (disaggregases) that can disaggregate the already formed aggregates (Parsell et al., 1994; Mogk et al., 1999; Doyle et al., 2013). Proteins recovered from aggregates are either refolded or degraded (Ravikumar et al., 2008; Douglas et al., 2009; Ciechanover and Kwon, 2015).

Yeast Hsp104 belonging to the Hsp100 family is a powerful AAA^+^ ATPase that has a hexameric ring structure with a central channel (Shorter, 2011; Torrente and Shorter, 2013). Once guided to protein aggregates by Hsp70, Hsp104 retrieves proteins from aggregates and threads them into nascent polypeptides (Seyffer et al., 2012; Lee et al., 2013; Lipinska et al., 2013; Carroni et al., 2014). During threading, Hsp70 and Hsp40 assist the unfolding of substrates to generate surface loops that are fed into the core of Hsp104 (Zietkiewicz et al., 2006). This disaggregation activity of Hsp104 was demonstrated to be effective for various aggregates (Mosser et al., 2004; Shorter and Lindquist, 2004; Arimon et al., 2008; Lo Bianco et al., 2008; DeSantis et al., 2012). Despite the disaggregase activities, Hsp104 exhibited the modest efficacy for the pathogenic misfolded proteins in human neurodegenerative diseases (DeSantis et al., 2012). The introduction of a few point mutations markedly increased its disaggregase activity for the preformed aggregates of α-synuclein in PD (Jackrel and Shorter, 2014), and TDP-43 and FUS in ALS (Jackrel and Shorter, 2014; Jackrel et al., 2014). Compared with wild-type Hsp104, the engineered form had increased ATPase activity with reduced dependence on the Hsp70/Hsp40 and, thus, exhibited enhanced activities in protein translocation and remodeling (Jackrel and Shorter, 2014; Jackrel et al., 2014).

In humans, Hsp70 and Hsp40 interact with the cochaperone Hsp110 to facilitate the disaggregation of protein aggregates (Gao et al., 2015; Nillegoda et al., 2015). Hsp110 belonging to the conserved Hsp70 superfamily has structural and functional similarity to Hsp70 including the nucleotide binding domain and acts as an NEF of Hsp70 (Polier et al., 2008). Although the action mechanism of the Hsp110-containing disaggregase complex remains unclear, it appears that the NEF activity of Hsp110 facilitates ADP release from Hsp70 (Rampelt et al., 2012). This ability to facilitate disaggregation *in vitro* has been equally observed with all three types of human Hsp110 isoforms: Hsp105α/HSPH1, Apg-2/HSPH2, and Apg-1/HSPH3 (Rampelt et al., 2012). The importance of Hsp110 in disaggregation has been demonstrates with various amyloids and prefibrillar oligomers and reactivate proteins from aggregates (Lo Bianco et al., 2008). Loss-of-function studies of Hsp110 have also shown its disaggregation activity in *C. elegans* (Rampelt et al., 2012), mouse cells (Yamagishi et al., 2010) and *Plasmodium falciparum* (Zininga et al., 2016).

## Protective role of chaperones in neurodegeneration

Many neurodegenerative diseases are directly caused by the excessive accumulation of misfolded proteins and their aggregates. During the pathogenesis, molecular chaperones play a central role in the refolding, degradation, and disaggregation of these pathogenic protein species. Extensive studies have shown that molecular chaperones promote the removal of pathogenic misfolded proteins and their aggregates.

### HD and other polyQ diseases

HD is a progressive neurodegenerative disease associated with the accumulation of mutant huntingtin (mHTT) that has the excessive repetition of glutamine residues, called polyQ, which causes misfolding (Shastry, 2003; Lee et al., 2011). These misfolded mHTT causes selective neuronal damage and death, leading to cognitive and motor defects (Gusella and MacDonald, 1998; Ramaswamy et al., 2007; Roos, 2010). Studies have shown that Hsp70 in a complex with Hsp40 plays a major role in inhibiting the formation of mHTT aggregates. Specifically, the Hsp70/Hsp40 machinery binds misfolded mHTT and holds its folding state to attenuate the formation of mHTT oligomers (Jana et al., 2000). The neuroprotective role of Hsp70 in the pathogenesis of PD is highlighted by a genetic screening of *Drosophila* PD model, which identified Hsp70 and Hsp40 as two major suppressors of the neurotoxicity caused by mHTT (Kazemi-Esfarjani and Benzer, 2000). Consistently, the knockout of Hsp70 in R6/2 transgenic HD mice has been shown to aggravate the symptoms in neurodegeneration (Wacker et al., 2009). A similar neuroprotective efficacy was observed with the neuronal chaperone HSJ1a (DNAJB2a) belonging to the Hsp40 family (Labbadia et al., 2012). In addition to the Hsp70-Hsp40 machinery, other members of the Hsp70 family have also been shown to counteract mHTT cytotoxicity. Specifically, the cytosolic chaperone Hsc70 binds and directly delivers mHTT to the lysosome via CMA, leading to selective degradation of mHTT and reduced toxicity (Bauer et al., 2010). This finding is further supported by *in vivo* studies using mice (Bauer et al., 2010) as well as flies (Gunawardena et al., 2003) overexpressing Hsc70. An ER counterpart of Hsp70, BiP/GRP78, has also been shown to counteract the accumulation of mHTT aggregates and apoptosis (Jiang et al., 2012). Besides the Hsp70 family members and their cochaperones, several other chaperones have been implicated in the refolding and/or degradation of polyQ protein aggregates, including Hsp84 (Mitsui et al., 2002), Hsp104 (Vacher et al., 2005), Hsp104/Hsp27 (Perrin et al., 2007), the chaperonin TRiC (Nollen et al., 2004; Behrends et al., 2006; Kitamura et al., 2006), and the cochaperone Prefoldin (Tashiro et al., 2013). Finally, HSPB7 belonging to small HSPs (Vos et al., 2010) and CHIP (Al-Ramahi et al., 2006) were shown to counteract the formation of polyQ aggregates in disease models.

### PD

PD is the second most common neurodegenerative disease after AD, affecting up to 10% of humans over 65 years. This protein misfolding disorder is associated with the loss of dopaminergic neurons in the substantia nigra pars compacta of brain (Wirdefeldt et al., 2011). PD is characterized by the formation of insoluble α-synuclein aggregates which are deposited as nuclear inclusions (Goedert, 2001; Ross and Poirier, 2004; Hasegawa et al., 2016) as a ubiquitinated form (Hasegawa et al., 2002). These inclusion, called Lewy bodies, are mainly composed of α-synuclein aggregates (Irizarry et al., 1998; Spillantini et al., 1998) together with various components of PQC, including Ub (Kuzuhara et al., 1988) and molecular chaperones such as Hsp70, Hsp90, Hsp60, Hsp40, Hsp27, and CHIP (McLean et al., 2002). This co-aggregation pattern indicates that α-synuclein aggregates deposited in Lewy bodies are the remnants that survived the attempts of molecular chaperones to maintain proteostasis. Specifically, Hsp70 recognizes the hydrophobic degron of misfolded α-synuclein through its substrate binding domain (Dedmon et al., 2005; Luk et al., 2008). By holding the folding status, Hsp70 facilitates the refolding of misfolded α-synuclein and inhibits the formation of its oligomers (Outeiro et al., 2008). The *in vivo* efficacy of Hsp70 was demonstrated with overexpressed Hsp70 in flies (Auluck and Bonini, 2002; McLean et al., 2004; Zhou et al., 2004; Opazo et al., 2008; Danzer et al., 2011) and mice (Klucken et al., 2004). Moreover, the depletion of molecular chaperones was shown to aggravate the degeneration of neurons caused by proteotoxicity (Ebrahimi-Fakhari et al., 2011).

The ER chaperone BiP belonging to the Hsp70 family can interact with α-synuclein and reduce its neurotoxicity (Gorbatyuk et al., 2012). Overexpressed BiP has been shown to protect nigral dopaminergic neurons in a rat model of PD, which correlates to reduced ER stress mediators and apoptosis (Gorbatyuk et al., 2012). The anti-aggregation and neuroprotective activity of BiP was further demonstrated with photoreceptor cells expressing aggregation-prone mutant rhodopsin (Gorbatyuk et al., 2010; Athanasiou et al., 2012). In addition to Hsp70 proteins, αB-crystallin belonging to small SHPs can interact with α-synuclein and inhibit the elongation of its fibrillar seeds by forming nonfibrillar aggregates (Kudva et al., 1997; Stege et al., 1999; Rekas et al., 2004; Shammas et al., 2011). Another small HSP, Hsp27, can arrest the aggregation of α-synuclein in the initial phage, perhaps by binding to the partially folded monomers (Rekas et al., 2007; Bruinsma et al., 2011).

### AD

AD is the most common neurodegenerative disorder caused by aggregation-prone proteins and selective loss, inactivation, or shrinkage in the mature nervous system (Regeur et al., 1994). The pathogenesis involves the deposit of amyloid-β (Aβ) both outside and inside the neurons as well as intracellular neurofibrillary tangles of hyperphosphorylated tau (Shankar et al., 2008; Honjo et al., 2012). Self-assembly of Aβ, which is not misfolded, generates its neurotoxic oligomers, which, in turn, grows into amyloid fibrils (Shankar et al., 2008; Honjo et al., 2012). Studies have shown that various molecular chaperones interact with intracellular Aβ species, which has been internalized by endocytosis, as well as tau and regulate their degradation. Specifically, αB-crystallin (HSPB5) and DnaJB6 bind to Aβ fibrils and inhibit their elongation and growth (Shammas et al., 2011; Mansson et al., 2014). In addition, Hsp70, Hsp40, and Hsp90 interact with the oligomer form of Aβ peptides (Evans et al., 2006). When overexpressed, Hsp70 and Hsp40 were shown to reduce the formation of Aβ aggregates and redirected it from growing into fibrillar to soluble circular structures (Evans et al., 2006). In contrast to Hsp70, Hsp90 supports the folding of tau and, thus, stabilizes this neurotoxic protein, facilitating tau pathology in AD model (Carman et al., 2013). The cochaperone BAG-1 forms a complex with Hsp70 and tau and can inhibit tau degradation in cultured cells, leading to the accumulation of both tau and APP (Elliott et al., 2007, 2009). Given the opposing roles of Hsp70 and Hsp90, the Hsp90 inhibitor 17-AAG was successfully used to induce the expression of various chaperones, such as Hsp70, Hsp40, and Hsp60 (Chen et al., 2014). The induction of these chaperones reduced Aβ toxicity in neurons (Chen et al., 2014). Another line of evidence supporting the protective role of chaperones in AD is provided by a study with UBB+1, a frameshift mutation of ubiquitin B (Hope et al., 2003). UBB+1 can inhibit the proteasome and, thus, can be deposited into intracellular protein inclusions in AD. The overexpression of UBB+1 induced the expression of HSPs, which, in turn, protected cells against oxidative stress.

### ALS

ALS is the most common adult onset motor neuron disease that affects the brainstem, cortex and spinal cord. It is characterized by the atrophy, weakness, and paralysis of muscles, leading to death within 3–5 years post diagnosis (Robberecht and Philips, 2013). The majority of ALS patients are sporadic, whereas 5–10% are familial, i.e., linked to mutations in specific genes. Numerous genetic mutations are linked to ALS, either genetically and/or pathologically. Amongst these, the mutations of SOD1, a free radical scavenger enzyme, accounts for 20% of familial ALS cases (Rosen et al., 1993). Several other ALS-linked mutated proteins form intracellular aggregates, including C9ORF72 (DeJesus-Hernandez et al., 2011), transactive response DNA binding protein (TDP-43), fused in sarcoma/translocated in liposarcoma (FUS), vesicle associated protein B (VAPB), ubiquilin-2, optineurin, and protein disulphide isomerase 1 and 3 (PDIA1 and PDIA3) (Robberecht and Philips, 2013). The majority of ALS cases are considered protein misfolding disorder because these mutations cause the accumulation of misfolded proteins and their aggregates.

Various molecular chaperones are implicated in the formation of these protein aggregates in ALS. For example, Hsp70/Hsp40, Hsp27, Hsp25, and αB-crystalline can form complexes with an ALS-causing mutant form of SOD, SOD1G93A. However, the overexpression of Hsp70 alone was not sufficient to reduce mutant SOD1 toxicity in ALS mouse model (Liu et al., 2005). Instead, PDI proteins exhibit a protective role in ALS models (Walker et al., 2010; Jeon et al., 2014). PDI assists the rearrangement of incorrectly arranged disulfide bonds of ER clients. It can also act as a chaperone that not only counteracts the aggregation of proteins independent of disulfide bonds but also delivers terminally misfolded proteins to ERAD (Quan et al., 1995). Over 15 missense mutations of PDIA1 and ERp57/PDIA3 were linked to ALS (Yang and Guo, 2016). Various *in vitro* and animal studies showed that PDI is deposited to the aggregates formed by the mutant forms of TDP-43 and FUS (Honjo et al., 2011; Farg et al., 2012), TDP-43 (Honjo et al., 2011; Walker et al., 2013), and VAPB (Tsuda et al., 2008). The overexpression of PDI reduces mutant SOD1 inclusions *in vitro* whereas PDI knockdown facilitates the formation of ALS inclusions (Walker et al., 2010).

Hsp27 also plays a protective role in the pathogenesis of ALS. Hsp27 binds mutant SOD1 *in vitro* and inhibits its fibril elongation (Yerbury et al., 2013). The overexpression of Hsp27 was shown to inhibit mutant SOD1-induced cell death (Patel et al., 2005). Hsp27 exhibited a synergistic efficacy when Hsp70 was coexpressed (Patel et al., 2005). In addition to Hsp27, HSJ1a shows a similar protective activity against the formation of mutant SOD aggregates at the late stage of the disease (Novoselov et al., 2013). HSJ1a interacts with SOD1G93 and facilitates its ubiquitination and proteasomal degradation.

## Therapeutic application targeting molecular chaperones in neurodegeneration

Given the protective role of molecular chaperones against pathogenic protein aggregates in neurodegenerative diseases, molecular chaperones are logical targets for drug development to modulate aggregation and clearance of the aggregates. Indeed, pharmaceutical induction of molecular chaperones has been demonstrated to effectively inhibit the formation of pathogenic aggregates in disease models.

Hsp90 supports in the folding/refolding and stability of a number of clients, including pathogenic misfolded protein aggregates in neurodegenerative diseases. While these activities are overall beneficial for refolding, however, Hsp90 also assists in the stability of neurotoxic proteins, favoring the accumulation of toxic protein aggregates (Schulte and Neckers, 1998; Boland et al., 2008; Eskelinen and Saftig, 2009; Chouraki and Seshadri, 2014). Therefore, one such strategy is the pharmaceutical inhibition of Hsp90. Geldanamycin competes with ATP and inhibits the folding and stabilization of neurotoxic proteins (Schulte and Neckers, 1998; Boland et al., 2008; Eskelinen and Saftig, 2009; Chouraki and Seshadri, 2014). In addition, upon binding to geldanamycin, Hsp90 releases a HSP-inducing transcript factor, HSF1 (McLean et al., 2004; Putcha et al., 2010). The dissociated HSF1 which otherwise would be sequestered by Hsp90 move to the nucleus and transcriptionally induces HSPs, such as Hsp70 (McLean et al., 2004; Putcha et al., 2010). Geldanamycin was successfully used to inhibit protein aggregation in the *Drosophila* (McLean et al., 2004; Putcha et al., 2010) and mouse PD model (Shen et al., 2005) and in a primary culture model of familial ALS (Batulan et al., 2006).

Despite its therapeutic efficacy, geldanamycin is toxic and cannot penetrate the blood brain barrier (BBB). A number of geldanamycin derivatives or the compounds that target HSF1 are now available, including geranylgeranylacetaone, celastrol, arimoclomol, withaferin A, 17-N-allylamino-17-demethoxygeldanamycin (17-AAG), and PU-DZ8 (Kieran et al., 2004; Niikura et al., 2006; Hoogstra-Berends et al., 2012; Khan et al., 2012; Kalmar et al., 2014; Sharma et al., 2015). Amongst these, celastrol is an anti-inflammatory and antioxidant compound extracted from a perennial creeping plant belonging to the Celastraceae family (Cleren et al., 2005). The treatment of celastrol in HD model mice resulted in the induction of Hsp70 expression associated with reduced loss of dopaminergic neurons induced by MPTP in the substantia nigra pars compacta (Cleren et al., 2005). Celastrol protected neurons against polyglutamine toxicity *in vivo* and *in vitro* (Zhang and Sarge, 2007) and reduced the β-amyloid level in mouse AD (Paris et al., 2010) and HD (Zhang and Sarge, 2007) models. BBB-permeable Hsp90 inhibitors, 17-AAG and PU-DZ8, were used to decrease the levels of phosphorylated tau in the AD model (Luo et al., 2007) and to inhibit neurodegeneration in a fly HD model (Fujikake et al., 2008). In addition, as Hsp90 inhibition causes undesirable proteotoxicity, HSF1A, a small benzyl pyrazole-based compound, has been developed to activate Hsf1 without inhibiting Hsp90 (Neef et al., 2010). Overall, studies using these HSP-inducing compounds in animal models of neurodegenerative diseases demonstrate that this strategy has potential for therapeutic application.

## Concluding remarks

Neurodegenerative diseases are caused by failure in PQC, which can be attributed to genetic mutations or alternatively an age-related decline in proteolytic activities. Molecular chaperones are an essential component of PQC in that they recognize unfolded or misfolded proteins, hold their folding status, and release them for spontaneous refolding. These nanoscale molecular machines can also facilitate the degradation of terminally misfolded proteins either through the UPS and autophagy. As the last defense mechanism of PQC, molecular chaperons can disaggregate the already formed aggregates. Thus, molecular chaperones play a pivotal role to protect neurons from the accumulation of pathogenic protein aggregates. It is therefore not surprising that pharmaceutical means are exploited to modulate the activities and functions of molecular chaperones. Indeed, small molecule compounds that target molecular chaperones such as Hsp90 have been successfully demonstrated to be effective in various neurodegenerative diseases. There is now an emerging consensus that proteostasis in diseases could be restored by using small molecule compounds or RNA interference that modulates chaperone expression or activities. A better understanding of chaperone functions in neurons will help the development of therapeutic means to restore proteostasis.

## Author contributions

All authors listed, have made substantial, direct and intellectual contribution to the work, and approved it for publication.

### Conflict of interest statement

The authors declare that the research was conducted in the absence of any commercial or financial relationships that could be construed as a potential conflict of interest.

## Abbreviations

17-AAG: 17-N-allylamino-17-demethoxygeldanamycin
Aβ: amyloid-β
AD: Alzheimer's disease
ALS: amyotrophic lateral sclerosis
BBB: blood brain barrier
C-domain: C-terminal dimerization domain
CHIP: C-terminus of Hsc70-interacting protein
CMA: chaperone-mediated autophagy
DUBs: deubiquitination enzymes
ERAD: ER-associated degradation
HD: Huntington's disease
Hsc70: heat shock cognate 70
HSF1: heat-shock factor 1
HSP: heat-shock protein
HSR: heat-shock response
M-domain: mid-domain
mHTT: mutant huntingtin
N-domain: N-terminal ATP-binding domain
NEF: nucleotide exchange factor
PD: Parkinson's disease
PDI: protein disulfide isomerase
PQC: protein quality control
RING: really interesting new gene
RIP-1: receptor interacting protein 1
SBD: substrate binding domain
TDP-43: transactive response DNA binding protein
TRiC: TCP-1 Ring Complex
TSE: transmissible spongiform encephalopathies
Ub: ubiquitin
UBL: Ub-like
UCHL5: ubiquitin C-terminal hydrolase L5
UPS: Ub-proteasome system
VAPB: vesicle associated protein B.
